# Orf Virus-Based Vectors Preferentially Target Professional Antigen-Presenting Cells, Activate the STING Pathway and Induce Strong Antigen-Specific T Cell Responses

**DOI:** 10.3389/fimmu.2022.873351

**Published:** 2022-05-09

**Authors:** Melanie Müller, Alena Reguzova, Markus W. Löffler, Ralf Amann

**Affiliations:** ^1^ Department of Immunology, Interfaculty Institute for Cell Biology, University of Tübingen, Tübingen, Germany; ^2^ Department of General, Visceral and Transplant Surgery, University Hospital Tübingen, Tübingen, Germany; ^3^ Department of Clinical Pharmacology, University Hospital Tübingen, Tübingen, Germany; ^4^ Cluster of Excellence iFIT (EXC2180) ‘Image-Guided and Functionally Instructed Tumor Therapies’, University of Tübingen, Tübingen, Germany

**Keywords:** parapoxvirus, ORFV, viral vector, vaccine, antigen-presenting cell, STING pathway, T cell response

## Abstract

**Background:**

Orf virus (ORFV)-based vectors are attractive for vaccine development as they enable the induction of potent immune responses against specific transgenes. Nevertheless, the precise mechanisms of immune activation remain unknown. This study therefore aimed to characterize underlying mechanisms in human immune cells.

**Methods:**

Peripheral blood mononuclear cells were infected with attenuated ORFV strain D1701-VrV and analyzed for ORFV infection and activation markers. ORFV entry in susceptible cells was examined using established pharmacological inhibitors. Using the THP1-Dual™ reporter cell line, activation of nuclear factor-κB and interferon regulatory factor pathways were simultaneously evaluated. Infection with an ORFV recombinant encoding immunogenic peptides (PepTrio-ORFV) was used to assess the induction of antigen-specific CD8+ T cells.

**Results:**

ORFV was found to preferentially target professional antigen-presenting cells (APCs) *in vitro*, with ORFV uptake mediated primarily by macropinocytosis. ORFV-infected APCs exhibited an activated phenotype, required for subsequent lymphocyte activation. Reporter cells revealed that the stimulator of interferon genes pathway is a prerequisite for ORFV-mediated cellular activation. PepTrio-ORFV efficiently induced antigen-specific CD8+ T cell recall responses in a dose-dependent manner. Further, activation and expansion of naïve antigen-specific CD8+ T cells was observed in response.

**Discussion:**

Our findings confirm that ORFV induces a strong antigen-specific immune response dependent on APC uptake and activation. These data support the notion that ORFV D1701-VrV is a promising vector for vaccine development and the design of innovative immunotherapeutic applications.

## Introduction

Orf virus (ORFV) is a large (~ 140 kb) double-stranded DNA (dsDNA) *Parapoxvirus* (1). The wildtype ORFV genome contains highly conserved regions comprising genes implicated in virulence and host immunomodulation (2). As a zoonotic pathogen ORFV primarily infects goats and sheep as its natural host. Humans are rarely affected and infections are typically associated with minor self-limiting symptoms (3, 4). The ORFV strain D1701, which was attenuated by successive *in vitro* passages in primary bovine and ovine cells, was used as a vaccine against Orf disease in its natural host animals, but it was discontinued due to lacking long-term protective effects (5, 6). Further *in vitro* passaging in Vero cells generated the Vero-adapted attenuated ORFV strain D1701-V, which lacks any pathogenic features, even in immunosuppressed sheep (7–10).

Further, substitution of the viral *vegf-e* gene by foreign transgenes generated the ORFV strain D1701-VrV. The poxviral early vegf promotor enables transgene expression without the requirement of viral replication, thus allowing ORFV to be used in non-permissive cells (10). ORFV D1701-VrV therefore represents an optimal viral vector candidate for animal and human use and possesses several desirable characteristics, including: 1.) a beneficial safety profile; 2.) a large genome, allowing for the integration of multiple/large genes; 3.) negligible induction of ORFV-specific immunity, allowing for repeated administration; 4.) potent induction of innate and adaptive immune responses against inserted transgenes; and 5.) large virus production capacity in cell cultures, facilitating the rapid generation of vaccine prototypes (11, 12). Meanwhile, recombinant ORFV D1701-VrV-based vaccines have been generated against several zoonotic pathogens, including avian influenza (13), rabies (11), pseudorabies (14), and Borna disease virus (15). Various animal models provided promising results, demonstrating protective immunity through respective vaccines with limited adverse effects (1, 16). Of note, recent studies with ORFV D1701-VrV by Reguzova et al. showed successful T cell induction against transgenes, while ORFV-specific epitopes remained unaffected (12, 17).

In spite of the emerging role as a vaccine vector and potential immunotherapeutic applications, the mechanisms by which ORFV D1701-VrV induces immunity, and effects observed in human cells, remain to be characterized. Respective knowledge is essential for the development of ORFV-based vaccine platforms and potential clinical applications for human use. Therefore, this study focused on the mechanisms involved in ORFV-induced immune responses in human cells. To this end, we sought to investigate: 1) the susceptibility of human primary lymphocytes to ORFV infection and the respective cell tropism; 2) the activation of peripheral blood mononuclear cells (PBMCs) subsets and antigen-presenting cells (APCs) after ORFV infection; 3) ORFV entry into susceptible cells; 4) the signaling pathway(s) involved after ORFV-infection of cells; and 5) induction of antigen-specific T cell responses through ORFV D1701-VrV *in vitro*.

## Materials and Methods

### Generation of ORFV D1701-V-D12-mCherry and ORFV D1701-V12-PepTrio-D12-Cherry

ORFV strain D1701-V-D12-mCherry (V-D12-Cherry) was generated as described previously (6, 10) to enable visualization of viral infection through the detection of mCherry florescence by flow cytometry. The novel artificial antigen “PepTrio”, designed specifically for use in this work, consists of three immunodominant epitopes from human cytomegalovirus (HCMV) proteins IE-1 (316-324; VLEETSVML) and pUL83/pp65 (495-503; NLVPMVATV and 120-128; MLNIPSINV), which all bind to the major histocompatibility complex (MHC) class I molecule HLA-A*02:01. The PepTrio-encoding gene (synthesized by Gene Art, Thermo Fisher Scientific, Waltham, MA, USA) was isolated as a *Bgl*II–*Eco*RI DNA fragment (164 bp) by agarose gel electrophoresis and Qiaex II gel extraction (Qiagen, Hilden, Germany) followed by ligation (Quick Ligation Kit, New England BioLabs, Frankfurt am Main, Germany) into *Bgl*II–*Eco*RI-digested pV12-Cherry (10). The resulting transfer plasmid, pV12-PepTrio, was used to transfect D1710-V-GFP-D12-mCherry-infected Vero cells using nucleofection (Cell Line V Nucleofector^®^ Kit, Lonza, Köln, Germany) to replace the GFP-encoding gene with PepTrio, as described previously (10). The new ORFV recombinant D1701-V12-PepTrio-D12-mCherry (V12-PepTrio-D12-Cherry, PepTrio-ORFV) was selected and purified by fluorescence-based negative selection, as described previously (10). PCR primers spanning the *vegf* locus [5’-GGTGCTCAGCGTGGTGGCGGTTTC-3’ (forward) and 5’-ACCACAAGGCCGCCCAGAAGACGCCGCTAG-3’ (reverse)] were commercially obtained (Metabion, Planegg, Germany) and were used to confirm the presence of a 738-bp amplicon for the PepTrio gene and the loss of an 1129-bp amplicon for the GFP-encoding gene. Sequencing confirmed the correct integration of the PepTrio gene into the *vegf* locus. The ORFV recombinants were purified, propagated, and titrated in Vero cells, as described previously (10).

### Donor Cells and Cell Lines

PBMCs were isolated from buffy coats obtained at the University Hospital Tübingen, Center for Clinical Transfusion Medicine, from HCMV-seronegative and HCMV-seropositive donors. The use of biomaterials was approved by the Ethics Committee of the Medical Faculty of Eberhard Karls University and the University Hospital of Tübingen (project number: 507/2017B01).

African green monkey kidney (Vero) cells (ATCC^®^) were cultured in Dulbecco’s Modified Eagle’s Medium (DMEM; Life Technologies) containing 5% fetal calf serum (FCS; Capricorn Scientific, Ebsdorfergrund, Germany) with 0.5% penicillin–streptomycin. All cells were cultured at 37°C with 5% CO_2_ in a humidified incubator.

### Cell Isolation and Macrophage and Dendritic Cell (DC) Differentiation

PBMCs were isolated from buffy coats by Ficoll density gradient centrifugation (Biocoll Separation Solution, Merck KGaA, Darmstadt, Germany). CD14+ monocytes were isolated from total PBMCs by magnetic cell sorting using CD14 microbeads (MACS, Miltenyi Biotec, Bergisch Gladbach, Germany). PBMCs or CD14+ monocytes were seeded at a density of 1×10^6^ or 1×10^5^ per well in a 96-well plate, respectively, and cultured with 200 µL Iscove’s Modified Dulbecco’s Medium (IMDM; Lonza, Köln, Germany) supplemented with 10% heat-inactivated FCS (Sigma-Aldrich, St. Louis, MO, USA). Monocyte-derived macrophages were differentiated from CD14+ cells with 50 ng/mL granulocyte-macrophage colony-stimulating factor (GM-CSF; Leukine^®^ (Sargramostim), Sanofi, Paris, France), immature dendritic cells (iDCs) were differentiated from CD14+ cells with 50 ng/mL GM-CSF and 50 ng/mL interleukin (IL)-4 (Peprotech, Rocky Hill, NJ, USA). Mature DCs (mDCs) were generated by stimulating iDCs with 100 ng/mL lipopolysaccharide (LPS; Sigma-Aldrich) for 24 h.

### Infection of Vero Cells With Virus Lysates From Infected APCs

DCs and macrophages were infected with V-D12-Cherry at MOI 1.0 and MOI 5.0 for 6 h, 24 h, and 96 h, respectively and lysed by repeated freezing and thawing. Vero cells were infected using DC- and macrophage-lysates (infection at MOI 5.0) with different dilutions for 24 h. The percentage of infected Vero cells was assessed by flow cytometry and viral infection was determined in respective samples, confirming a linear range of infection from 0.5-30%.

### Monocyte Depletion

Monocytes were depleted from total PBMCs using CD14 microbeads and depletion columns (MACS) in accordance with the manufacturer’s instructions. Successful depletion (98-100%) was verified by flow cytometry.

### Viral Infection

PBMCs, monocytes, macrophages and DCs were inoculated with V-D12-Cherry for *in vitro* infection at a multiplicity of infection (MOI) of 5.0 for the indicated times, if not specifically indicated otherwise.

### Inhibition of Phagocytosis and Macropinocytosis

Phagocytosis was inhibited by incubating cells with 20 µM cytochalasin D (Sigma-Aldrich) for 30 min before infection. Macropinocytosis was inhibited by incubating cells with 3 µM rottlerin (Sigma-Aldrich) for 30 min before infection.

### Cytometric Bead Array

For analysis of CXCL10 production, DCs were infected with V-D12-Cherry for 24 h (MOI 5.0). Quantification of CXCL10 in the supernatants of ORFV-infected DCs was performed using BD™ Cytometric Bead Array (CBA) (BD Biosciences, Franklin Lakes, NJ, USA) according to the manufacturer’s instructions.

### Activation of THP1-Dual™ Cells

THP1-Dual™ cells, THP1-Dual^™^ KO-IFNAR2 cells, THP1-Dual^™^ KO-MyD88 cells, and THP1-Dual™ KO-STING cells were purchased (InvivoGen, San Diego, CA, USA). Secreted embryonic alkaline phosphatase (SEAP) and luciferase activity were assessed after infection with V-D12-Cherry (MOI 10.0) in accordance with the manufacturer’s protocol for THP1-Dual™ cells. Infection rates were determined by flow cytometry and established at 25% ± 5%.

### Memory T Cell Expansion

A total of 5×10^6^ PBMCs per well, obtained from HCMV-seropositive blood donors, were seeded into a 24-well plate in 2 mL medium (RPMI-1640 with 10% heat-inactivated FCS) and incubated for 6 h. PBMCs were infected with PepTrio-ORFV or V-D12-Cherry (mock virus control) at the indicated MOI, or stimulated with 1 µg/mL HCMV pp65 _495–503_ NLVPMVATV peptide (positive control) for CD8+ T cell activation. Every 2–3 days, 500 μL of medium was replaced with 500 μL fresh medium containing 20 U/mL IL-2 (R&D Systems, Minneapolis, MN, USA). After 12 days of stimulation, analysis of T cell expansion and antigen-specific T cell functionality was performed by HLA-tetramer and intracellular cytokine staining, respectively.

### Priming of Naïve T Cells

A total of 1×10^8^ PBMCs from HCMV-seronegative blood donors were used to isolate CD14+ monocytes and to subsequently induce differentiation into DCs as described above. DCs (2×10^5^) were seeded into a 96-well pate in 200 µL medium per well. After one week, DCs were infected with PepTrio-ORFV (MOI 5.0) for 6 h or stimulated with 10 ng/mL IL-4 (Peprotech), 800 U/mL GM-CSF, 10 ng/mL LPS, and 100 U/mL IFN-γ (Peprotech) for 24 h and loaded with 25 μg/mL HCMV pp65 _495−503_ NLVPMVATV peptide for 2 h. Isolation of CD8+ T cells from cryopreserved PBMCs was performed by magnetic cell sorting using CD8 microbeads (MACS). Subsequently, 1×10^6^ CD8+ T cells were added to the infected or peptide-loaded DCs in the presence of 5 ng/mL IL-12 (PromoCell, Heidelberg, Germany). Every 2–3 days, 100 µL medium was replaced with 100 µL fresh medium containing 40 U/mL IL-2. After one week of co-culture of DCs and CD8+ T cells, the latter were restimulated with autologous peptide-loaded PBMCs. For this purpose, autologous PBMCs were thawed and loaded with 25 μg/mL HCMV pp65 _495–503_ NLVPMVATV peptide and incubated for 2 h. A total of 1×10^6^ peptide-loaded PBMCs were added to each well following irradiation (30 Gy). T cells were stimulated three or four times in total with 7-day intervals. One week after the last re-stimulation, analysis of antigen-specific CD8+ T cell responses was performed by HLA-tetramer staining.

### Antibody Staining and Flow Cytometry

To prevent non-specific antibody binding, cells were treated with Fc block (BioLegend, San Diego, CA, USA) before antibody staining according to the manufacturer’s instructions. To stain for viability, cells were washed with PBS and stained using the Zombie Aqua™ Fixable Viability Kit (BioLegend). Before extracellular staining, cells were washed twice with staining buffer (2 mM ethylenediaminetetraacetic acid [EDTA], 2% FCS, and 0.02% NaN_3_ in PBS).

To analyze PBMC activation, cells were stained with antibodies specific for CD4^PacificBlue^, CD8^APC/Cy7^, CD14^AlexaFluor700^, CD19^PerCP^, CD56^BV605^, and CD69^PE^ (all BioLegend). Infected monocytes were stained for CD14^AlexaFluor700^ and the activation marker human leukocyte antigen (HLA)-DR^BV711^. Monocyte-derived macrophages were stained for CD14^AlexaFluor700^, the activation markers HLA-DR^BV711^, CD80^FITC^, CD86^BV605^, and CD40^PE/Cy7^ (all BioLegend). Monocyte-derived DCs were stained for CD11c^BV421^, the activation markers HLA-DR^BV711^, CD80^FITC^, CD86^BV605^, and CD40^PE/Cy7^, and the maturation marker CD83^APC^ (all BioLegend). All staining steps were performed by incubating the cells and antibodies at 4°C for 30 min followed by two washing steps with staining buffer.

For HLA-tetramer staining, cells were washed twice with 200 μL PBS and then resuspended in 50 μL tetramer buffer (50% FCS, 2 mM EDTA in PBS) with 1 µL phycoerythrin (PE)-conjugated HLA-tetramer. Incubation was performed at room temperature for 30 min in the dark. After incubation, cells were stained for viability (Zombie Aqua™ Fixable Viability Kit) and for extracellular markers using antibodies specific for CD4^PacificBlue^ and CD8^APC/Cy7^ (BioLegend).

For intracellular cytokine staining, expanded CD8+ memory T cells were stimulated with HCMV pp65 _495–503_ peptide NLVPMVATV (1 µg/mL) in the presence of 10 μg/mL brefeldin A (Sigma-Aldrich) for 16 h. After incubation, cell viability staining was performed (Zombie Aqua™ Fixable Viability Kit), followed by cell surface staining with antibodies specific for CD4^PacificBlue^ and CD8^APC/Cy7^ (BioLegend). The cells were subsequently fixed and permeabilized using BD Cytofix/Cytoperm (BD Biosciences, Franklin Lakes, NJ, USA) at 4°C for 30 min and incubated with antibodies specific for IFN-γ^APC^ and tumor necrosis factor (TNF)^PE/Cy7^ (BioLegend).

Samples were analyzed using a LSR Fortessa™ flow cytometer (BD Biosciences) and data processing was performed with FlowJo^®^ software (TreeStar Inc., Ashland, OR, USA).

### Statistical Analysis

All statistical analyses were performed using GraphPad Prism 9 software. Data are presented as means ± standard deviation. Normality of data was tested using the Shapiro-Wilk test. Comparisons between groups were performed using a two-tailed Student´s *t*-test. A p < 0.05 was considered to indicate statistical significance.

## Results

### ORFV Preferentially Targets Antigen-Presenting Cells *In Vitro*

**Figure 1:**
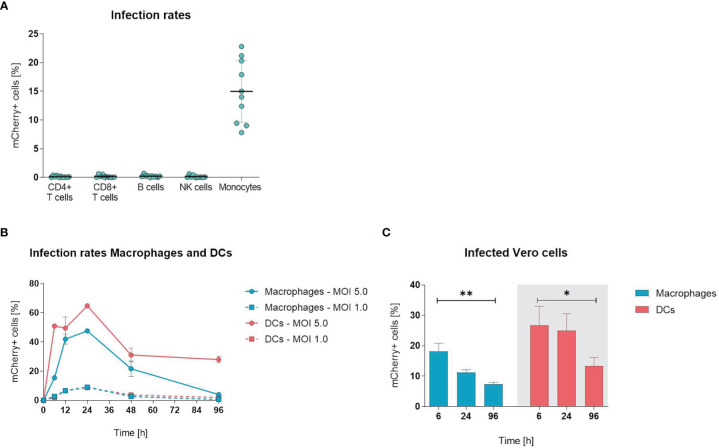
Orf virus (ORFV) preferentially targets antigen-presenting cells *in vitro*. **(A)** Human peripheral blood mononuclear cells (PBMCs) were inoculated with V-D12-Cherry [multiplicity of infection (MOI) 5.0, 24 h]. Different cell subsets were identified by extracellular staining [CD4: CD4+ T cells, CD8: CD8+ T cells, CD19: B cells; CD56: natural killer (NK) cells; CD14: monocytes] and the percentage of viable mCherry+ cells was determined by flow cytometry analysis. Data represent mean ± SD of biological replicates. **(B)** Macrophages and DCs were infected with V-D12-Cherry (MOI 1.0 and MOI 5.0). The percentage of viable mCherry+ cells was determined at 6, 12, 24, 48 and 96 h post-infection by flow cytometry. X-axis: time post-infection (h); Y-axis: percentage of mCherry+ cells. Data represent mean ± SD of technical replicates (n=3). **(C)** Cell lysate of V-D12-Cherry-infected (MOI 5.0) macrophages and DCs at 6 h post-infection (hpi), 24 hpi, and 96 hpi was used to infect Vero cells. The percentage of mCherry+ Vero cells was determined 24 hpi. X-axis: time point after macrophage or DC infection; Y-axis: percentage of mCherry+ Vero cells. Data represent mean ± SD of technical replicates (n=3) and are representative for 3 independent experiments. Statistical differences are shown (unpaired *t*-test). *P < 0.05; **P < 0.01.

To examine whether ORFV infects and drives the expression of transgenes in leukocytes, PBMCs were infected with ORFV encoding mCherry (V-D12-Cherry) for 24 h *in vitro*, stained and assessed by flow cytometry to identify cell subsets (T cells, B cells, NK cells and monocytes) susceptible for infection. Expression of mCherry is driven by an early poxviral promoter that enables strong early transgene expression without the need for ORFV genome replication or infectious virus production. After infection, mCherry expression, indicating viral infection, remained restricted to CD14+ monocytes and resulted in a mean infection rate of ~15% (**Figure 1A**). All other assessed cell populations were unaffected.

Since ORFV incubation with PBMCs only infected monocytes, we next analyzed whether other APCs (*i.e*., macrophages and DCs) were also susceptible for infection. When infecting monocyte-derived macrophages and DCs with ORFV for different periods, the percentage of mCherry-positive macrophages and DCs increased and peaked at 24 h, subsequently decreasing until 96 h (**Figure 1B**).

Next, we determined whether ORFV replicates in human APCs. This was investigated by assessing the percentage of Vero cells that were infected through incubation with cell lysates from macrophages and DCs previously infected with ORFV and then cultured for different periods of time.

The infection rates of Vero cells lay in the linear range from 0.5 – 30% of infected cells at 24 hours post infection. Significantly fewer Vero cells became infected when incubated with cell lysates from both macrophages and DCs harvested 96 h after ORFV incubation as compared to incubation with respective cell lysates harvested earlier at 6 h (**Figure 1C**), suggesting that after 96 h less ORFV virus is present in the APC lysate than after 6 h. Thus, the decreasing amount of virus over time indicates a lack of productive generation of infectious virus particles in APCs.

Overall, these results demonstrate that professional APCs are susceptible to ORFV infection and enable expression of the transgene without the need for viral replication.

### ORFV Is Taken Up by Professional APCs *via* Macropinocytosis

**Figure 2:**
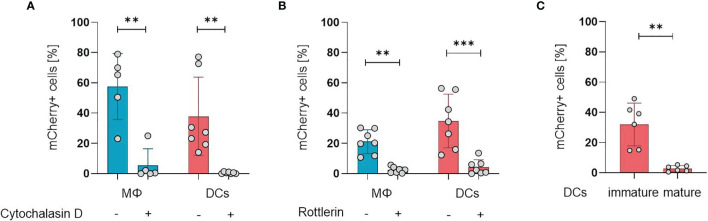
ORFV is taken up by professional APCs *via* macropinocytosis. **(A)** Macrophages (MΦ) and DCs were treated with 20 µM cytochalasin D or left untreated. After infection with V-D12-Cherry (MOI 5.0, 24 h) the level of mCherry expression was measured using flow cytometry. The percentage of positive cells is indicated. **(B)** Macrophages and DCs were treated with 3 µM rottlerin or left untreated. After infection with V-D12-Cherry (MOI 5.0, 24 h) mCherry expression was measured using flow cytometry. The percentage of positive cells is indicated. **(C)** Immature DCs and LPS-matured DCs were infected with V-D12-Cherry (MOI 5.0, 24 h). The percentage of mCherry+ cells was determined using flow cytometry. Of note: cells of different donors were used for experiments shown in **(A–C)**. Data represent mean ± SD of biological replicates. Statistical differences are shown (paired *t*-test). **P < 0.01; ***P < 0.001.

After establishing the susceptibility of APCs for ORFV infection, we aimed to investigate the mechanisms of ORFV entry into these cells. APCs take up pathogens either *via* receptor-mediated endocytosis (*e.g*., phagocytosis) or *via* receptor-independent macropinocytosis, but both processes require actin polymerization (18, 19). Therefore, we inhibited actin polymerization in monocyte-derived macrophages and DCs by cytochalasin D before ORFV infection. Treated APCs remained negative for ORFV infection, while untreated APCs became infected (p < 0.01; **Figure 2A**), indicating that actin cytoskeleton rearrangement is required for ORFV uptake.

To differentiate an uptake *via* phagocytosis from macropinocytosis, the cells were treated with the protein kinase C delta inhibitor rottlerin that specifically inhibits macropinocytosis at low concentrations (20, 21). The infection rate in DCs after treatment was significantly reduced compared to controls (p < 0.001), however, a baseline infection was discernible in the latter (**Figure 2B**), confirming that ORFV mainly enters DCs *via* macropinocytosis. Respective findings could also be shown in macrophages (**Figure 2B**).

While phagocytosis and macropinocytosis are well-known uptake mechanisms of immature DCs, this ability is lost during DC maturation and activation (22). Therefore, we tested whether DC maturation influences the uptake of ORFV. Both immature DCs (iDCs) and matured DCs (mDCs) were incubated with ORFV. Results confirmed that iDCs were significantly more frequently infected than mDCs (p < 0.001; **Figure 2C**).

### PBMCs and Professional APCs Are Activated After ORFV Infection *In Vitro*

**Figure 3:**
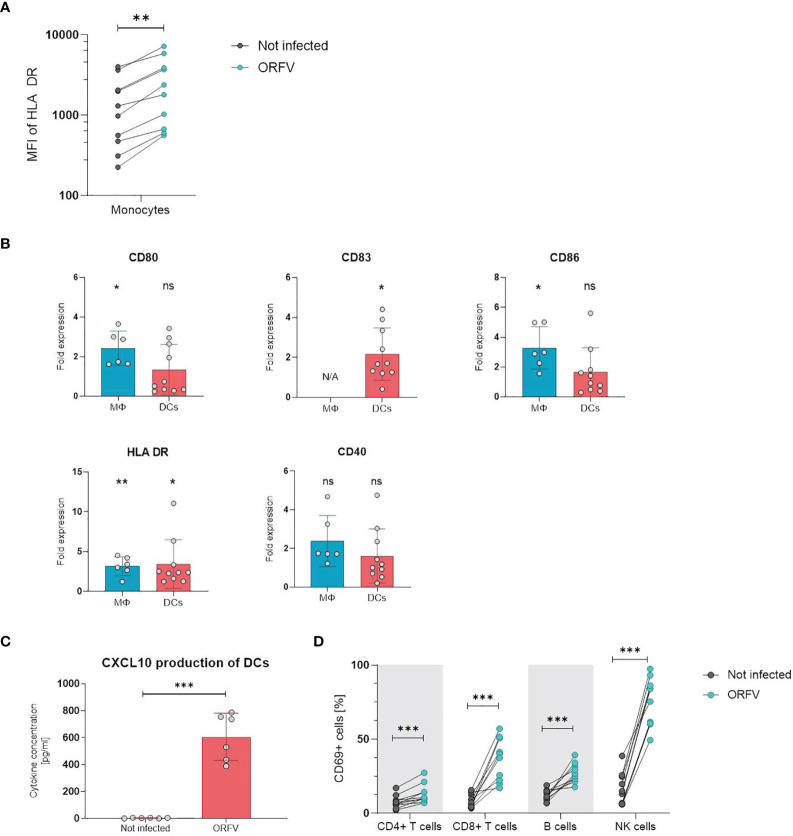
PBMCs and professional APCs are activated after ORFV infection *in vitro.* **(A)** Monocytes were inoculated with V-D12-Cherry (MOI 5.0, 24 h). Expression of human leukocyte antigen (HLA)-DR after infection was determined by flow cytometry. Expression of the activation marker HLA-DR on monocytes is depicted as the specific mean fluorescent intensity (sMFI) calculated as: MFI of the sample *minus* MFI of the isotype control. The change in HLA-DR expression was compared between non-infected and V-D12-Cherry-infected monocytes. Statistical differences are shown (paired *t*-test). **P < 0.01. **(B)** Macrophages (MΦ) and DCs were infected with V-D12-Cherry (MOI 5.0, 24 h). The expression of surface markers CD80, CD83, CD86, HLA-DR, and CD40 was analyzed between non-infected and V-D12-Cherry-infected cells and is indicated as the fold expression compared to expression on non-infected cells. Data represent mean ± SD of biological replicates. Statistical differences are shown for each marker (one sample *t*-test). ns, not statistically significant; *P < 0.05; **P < 0.01. **(C)** DCs were infected with V-D12-Cherry (MOI 5.0, 24 h). The concentration of CXCL10 in cell culture supernatant was determined *via* Cytometric Bead Array (CBA). Statistical differences are shown (paired *t*-test). ***P < 0.001. **(D)** Human peripheral blood mononuclear cells (PBMCs) were inoculated with V-D12-Cherry (MOI 5.0, 24 h). Expression of the activation marker CD69 after infection was determined by flow cytometry. The percentage of T cells, B cells, and NK cells expressing the early activation marker CD69 is indicated. Statistical differences are shown for all analyzed cell subsets (paired *t*-test). ***P < 0.001.

Next, we examined whether incubation with ORFV induces activation of leukocytes *in vitro*. First, we analyzed the activation state of APCs upon ORFV uptake. To characterize monocyte activation, CD14+ cells among PBMCs were purified and stained for HLA-DR 24 h after ORFV infection. Results revealed that HLA-DR expression was significantly increased in the ORFV-infected monocytes when compared to untreated controls (p < 0.01; **Figure 3A**).

To investigate activation of professional APCs after ORFV-infection, macrophages and DCs were incubated with ORFV for 24 h and subsequently expression of CD80, CD86, HLA-DR, and CD40 were assessed on CD14+ macrophages and CD11c+ DCs. DCs were further examined for the maturation marker CD83 (23). The expression of activation markers CD80, CD86 and HLA-DR was strongly increased on macrophages following infection compared to the non-infected control. The expression of all assessed activation markers observed on DCs from different donors varied substantially. However, the proportion of HLA-DR- and CD83-positive DCs were found markedly increased following infection (**Figure 3B**). In addition, analysis of cell culture supernatant 24 h after ORFV infection showed that DCs produced a significantly increased amount of CXCL10 after incubation with ORFV compared to uninfected DCs (**Figure 3C**). To investigate the effect of APC activation on activation of lymphocytes, we next analyzed the activation state of cell subsets in human PBMCs after incubation with ORFV *in vitro*. Following incubation with ORFV a significantly increased percentage of CD4+ and CD8+ T cells, B cells as well as NK cells expressed CD69, as compared to untreated controls (all p < 0.001; **Figure 3D**). This activation state was found particularly pronounced in NK cells.

These results indicate that infection with ORFV induces activation of APCs upon virus uptake and subsequently leads to the activation of T cells, B cells, and NK cells.

### Lymphocyte Activation Depends on ORFV-Infected Monocytes

**Figure 4:**
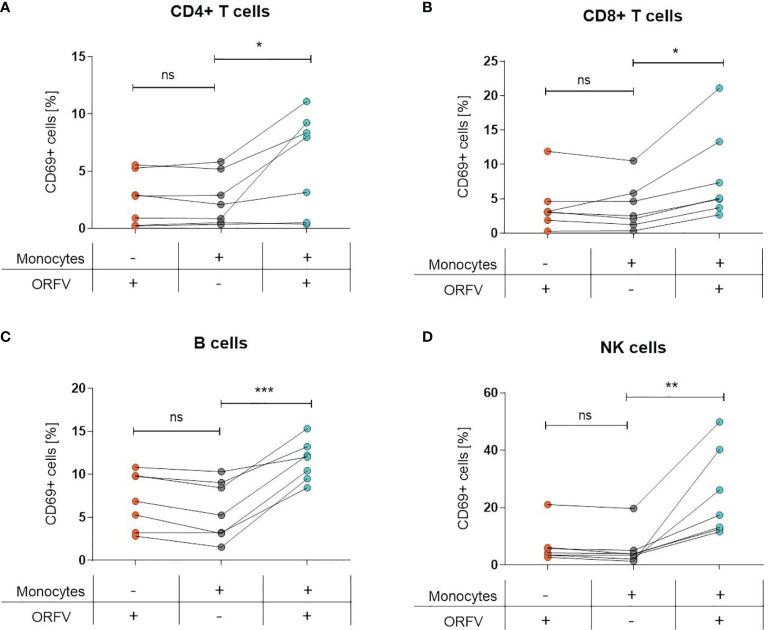
Lymphocyte activation is dependent on ORFV-infected monocytes. PBMCs and monocyte-depleted PBMCs were infected with V-D12-Cherry (MOI 5.0, 24 h) and stained with a specific antibody against the early activation marker CD69. The percentage of CD69+ cells is indicated. The change in CD69 expression was compared between non-infected and V-D12-Cherry-infected PBMCs, as well as V-D12-Cherry-infected monocyte-depleted PBMCs. Statistical differences (paired *t*-test) are shown for **(A)** CD4+ T cells, **(B)** CD8+ T cells, **(C)** B cells, and **(D)** NK cells. ns, not statistically significant; *P < 0.05; **P < 0.01; ***P < 0.001.

Since infection of PBMCs with ORFV led to an increase of CD69+ cells among lymphocytes, we sought to investigate the role of monocytes in the activation of other cell subsets. Thus, both total PBMCs and monocyte-depleted PBMCs were infected with ORFV and assessed for CD69 expression. While the infection of total PBMCs led to the activation of the different cell subsets, as evidenced before, the proportion of CD69-expressing CD4+ and CD8+ T cells, B cells, or NK cells among lymphocytes was not increased when monocytes were depleted (**Figures 4A–D**).

These data suggest that uptake of ORFV by APCs is a prerequisite for subsequent lymphocyte activation.

### Activation of the Stimulator of Interferon Genes (STING) Pathway in APCs Through ORFV Infection

**Figure 5:**
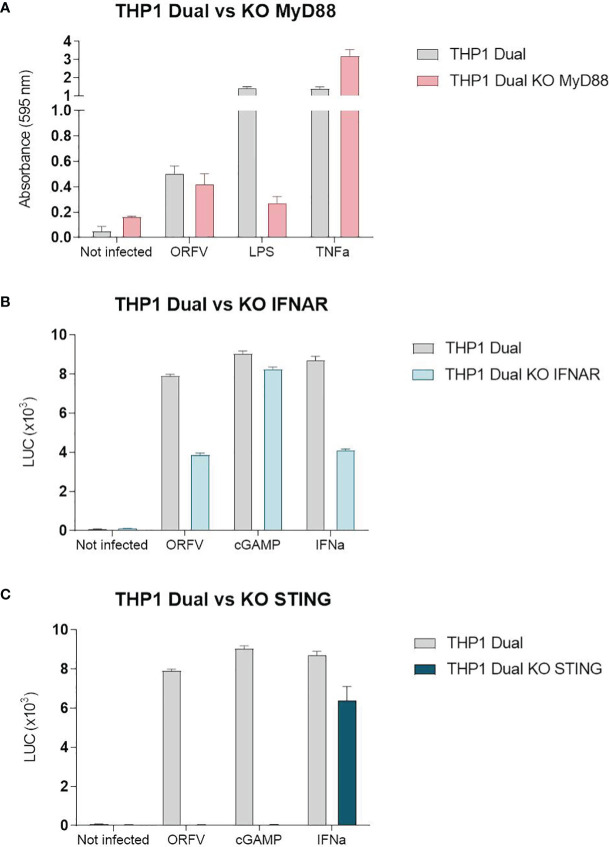
The stimulator of interferon genes (STING) pathway is required for the activation of THP1-Dual cells. **(A)** THP1-Dual cells and THP1-Dual KO myeloid differentiation primary response 88 (MyD88) cells were infected with V-D12-Cherry (MOI 10.0, 24 h). Secreted embryonic alkaline phosphatase (SEAP) was analyzed in the cell culture supernatant. LPS stimulation (100 ng/ml) and tumor necrosis factor (TNF) stimulation (100 ng/ml) served as positive controls. Non-infected cells served as a negative control. **(B)** THP1-Dual cells and THP1-Dual KO interferon alpha receptor cells (IFNAR) were infected with V-D12-Cherry (MOI 10.0, 24 h). Luciferase secretion was analyzed in the cell culture supernatant. cGAMP (10 µ/ml) and interferon (IFN)-α stimulation (10^6^ U/ml) served as a positive control. Non-infected cells served as a negative control. **(C)** THP1-Dual cells and THP1-Dual KO STING cells, were infected with V-D12-Cherry (MOI 10.0, 24 h). Luciferase secretion was analyzed in the cell culture supernatant. cGAMP (10 µ/ml) and interferon (IFN)-α stimulation (10^6^ U/ml) served as a positive control. Non-infected cells served as a negative control. SEAP and luciferase activity was assessed in cells with infection rates of 25% ± 5%. Data represent mean ± SD of technical replicates (n=3) and are representative for 3 independent experiments.

To investigate ORFV-mediated APC activation in more detail, a monocytic reporter cell line (THP1-Dual cells) was used to investigate pathways involved in monocyte activation. These cells allow for simultaneous studies of the NF-κB and interferon regulatory factor pathways through SEAP and luciferase activity, respectively. To assess pathways required for APC activation, THP1-Dual reporter cells were infected with ORFV. Infected THP1-Dual KO MyD88 cells exhibited comparable SEAP production as infected THP1-Dual control cells (**Figure 5A**), indicating that ORFV-mediated APC activation occurs independent of MyD88. Luciferase activity in infected THP1-Dual KO IFNAR cells was less compared to Luciferase activity in THP1-Dual cells (**Figure 5B**), suggesting that ORFV-mediated APC activation occurs partially dependent of IFNAR2. However, infection of THP1-Dual KO STING cells failed to induce luciferase activity, indicating that the activation of APCs by ORFV is mediated by the STING pathway (**Figure 5C**).

### Induction of Cellular Immune Responses *In Vitro*

**Figure 6:**
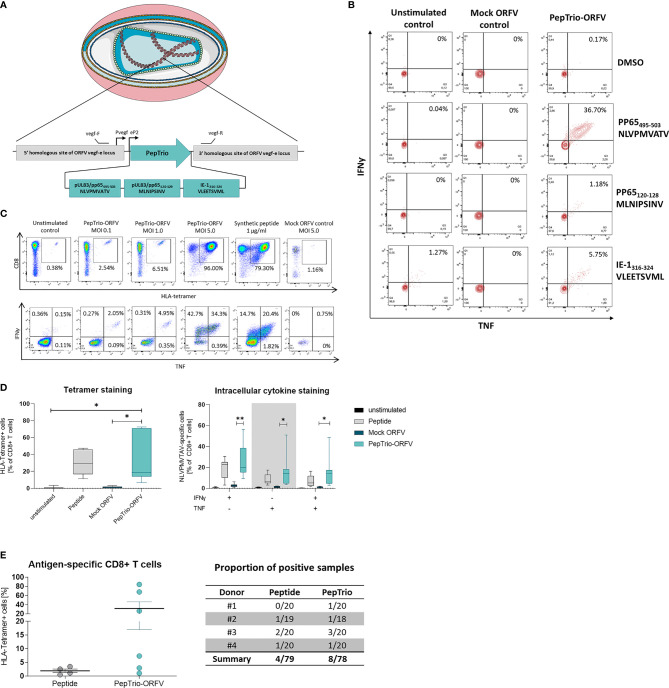
PepTrio-ORFV induces robust antigen-specific CD8+ T cell responses *in vitro*. **(A)** Schematic illustration of PepTrio integrated into the ORFV genome. PepTrio consists of minigenes that encode the HLA-A*0201 restricted epitopes HCMV pp65 _495−503_ NLVPMVATV, pp65 _120−128_ MLNIPSINV, and IE-1 _316−324_ VLEETSVML. Each epitope is encoded with its own start and stop codon. Pvegf and eP2 denote the early promoters, and vegf forward and vegf reverse indicate the primer-binding locations. **(B)** PBMCs from human cytomegalovirus (HCMV)-seropositive blood donors were presensitized for 12 days with Mock-ORFV or PepTrio-ORFV (MOI 5.0) or kept unstimulated. Activation of HCMV pp65 NLVPMVATV-, pp65 MLNIPSINV- and IE-1 VLEETSVML-specific CD8+ cells upon stimulation was determined by intracellular cytokine staining. **(C, D)** PBMCs from human cytomegalovirus (HCMV)-seropositive blood donor were presensitized for 12 days with PepTrio-ORFV or Mock-ORFV at the indicated MOIs, or kept unstimulated. Synthetic HCMV pp65 _495–503_ NLVPMVATV peptide was used as a control. Activation of HCMV pp65 NLVPMVATV-specific CD8+ cells upon stimulation was determined by HLA-tetramer staining and subsequent intracellular cytokine staining. **(D)** Data represent mean ± SD of biological replicates (n=7). Statistical differences (paired *t*-test) are shown. *P < 0.05; **P < 0.01. **(E)** Human monocyte-derived DCs of 4 donors were exposed to PepTrio-ORFV (MOI 5.0) for 6 h, followed by co-cultivation with autologous CD8+ T cells. After 4 weeks, the activation of naïve CD8+ T cells was assessed by HLA-tetramer staining. Peptide-loaded DCs used to stimulate CD8+ T cells served as a control.

We assessed whether ORFV-infected APCs present encoded HLA class I-restricted epitopes leading to the activation of antigen-specific T cells. To this end, a recombinant ORFV encoding the artificial antigen PepTrio (V12-PepTrio-D12-Cherry; abbreviated as PepTrio-ORFV) was used, encoding three HLA-A*0201 restricted epitopes from cytomegalovirus (HCMV), i.e. pp65_495−503_ NLVPMVATV, pp65_120−128_ MLNIPSINV, and IE-1_316−324_ VLEETSVML (**Figure 6A**). First the activation and expansion of antigen-specific memory CD8+ T cells with different antigen specificities was assessed. Therefore, PBMCs from a HCMV-seropositive donor were infected with PepTrio-ORFV (MOI 5.0) and the frequency of transgene-specific CD8+ T cells was determined by flow cytometry after 12 days. PBMC stimulation with PepTrio-ORFV activated a robust peptide-specific CD8+ T cell recall response against all encoded epitopes (**Figure 6B**).

Further, the relation between ORFV infection dose and the proliferation and activation of memory CD8+ T cells was investigated. Again, PBMCs from a HCMV-seropositive donor were infected with PepTrio-ORFV at different MOIs (0.1, 1.0 or 5.0). The frequency of HCMV pp65_495−503_-specific CD8+ T cells was assessed 12 days after ORFV infection by flow cytometry using HLA-tetramer staining as well as intracellular cytokine staining for TNF and IFN-γ. Thereby, antigen-specific CD8+ T cell recall responses could be shown as depending on the ORFV infection dose (**Figure 6C**). In summary, proliferation and activation of memory CD8+ T cells was demonstrated in PBMCs of 7 individual HCMV-seropositive donors. Overall, infection with PepTrio-ORFV (MOI 5.0) resulted in the highest frequency of HCMV pp65_495−503_-specific CD8+ T cells (**Figure 6D**).

Finally, priming of naive CD8+ T cells with PepTrio-ORFV-encoded peptides was also assessed *in vitro*. HLA-tetramer staining showed that HCMV pp65_495−503_-specific CD8+ T cells could be successfully induced by ORFV-infected DCs (**Figure 6E**). Thereby, successful priming with ORFV-infected DCs was shown more frequently than when using peptide-loaded DCs.

Altogether, these data demonstrate that APCs infected with PepTrio-ORFV enable priming of naïve CD8+ T cells and induce the activation of functional memory CD8+ T cells against encoded HLA class I-restricted epitopes.

## Discussion

ORFV D1701-VrV is a promising viral vector both for the development of novel vaccines and innovative immunotherapeutic approaches, enabling potent antigen-specific immune responses. Details on the precise mechanisms by which ORFV activates immune responses remain to be elucidated. The presented findings now demonstrate that ORFV-mediated T cell activation depends on the uptake of ORFV by APCs and on their activation *via* the STING pathway.

Analysis of cell tropism of ORFV revealed that only professional APCs such as monocytes, macrophages and DCs were shown to be susceptible to ORFV infection. In contrast, infection of T cells, B cells, and NK cells was neither detected in PBMCs nor in monocyte-depleted PBMCs. Experiments with rabbit PBMCs recently indicated this ORFV tropism. After infection of isolated rabbit PBMCs with D1701-V-Cherry, expression of mCherry was observed only in monocytes (24). The preferential targeting of professional APCs has also been observed in the context of other members of the *Poxviridae* family. In 2011, Flechsig et al. showed the preferential infection of monocytes when elucidating the cell tropism of modified vaccinia Ankara virus (MVA) in human PBMCs (25) and later confirmed by Altenburg et al. (26). In a large-scale study, cell tropism of MVA was investigated *in vitro* in human PBMCs, *ex vivo* in murine lung explants, and *in vivo* in mice, ferrets, and macaques after intranasal droplet infusion and following intramuscular injection. Regardless of the experimental set-up, animal model, or route of administration, the preferential infection of professional APCs was demonstrated.

By infecting professional APCs for different periods, we observed an increasing percentage of mCherry-positive cells until 24 h after infection, which subsequently decreased. Since infection of APCs was not synchronized, the observed increase of infected cells over the first 24 h was expected. Considering that infectious ORFV particles are produced considerably later than it is known for other poxviruses (27, 28), these results indicate the lack of viral spread in APCs. The absence of productive infectious virus particle generation in APCs was further demonstrated by incubating Vero cells with cell lysates from macrophages and DCs harvested 6 h and 96 h post infection. Moreover, since mCherry expression is driven by an early poxviral promoter, these results prove that expression of a transgene by ORFV is possible despite abortive infection.

Using established pharmacological inhibitors that prevent the entry of ORFV into APCs, we could determine entry *via* macropinocytosis as a main route. This is in line with other members of the *Poxviridae* family. For example, Sangren et al. demonstrated that vaccinia virus enters monocyte-derived DCs *via* macropinocytosis (29). Nevertheless, phagocytosis and other forms of endocytosis may still represent ancillary forms of entry (30). Our observations that following maturation, APCs lose the ability to take up ORFV conforms with results from Sallusto et al., who found that DC maturation led to an irreversible loss of macropinocytosis (20). It has been suggested that this loss in antigen capture and processing capacity may be required for optimal antigen presentation to T cells (20).

Previous studies have investigated the activation of APCs by ORFV strain D1701-B, the original strain from which ORFV strain D1701-VrV was derived. After infection of murine bone marrow-derived DCs, increased expression of MHC class I and class II as well as CD86 was shown (31, 32). In this study, enhanced expression of the co-stimulatory molecules CD80, CD86, and CD40 as well as the MHC class II molecule HLA-DR and the production of CXCL10 successfully demonstrated the activation of human APCs by ORFV strain D1701-VrV for the first time. It was further shown that infection and activation of APCs is required for subsequent activation of T cells, B cells, and NK cells. A similar dependence was also demonstrated in studies conducted with MVA. Using an *in vivo* mouse model, Liu et al. demonstrated that MVA-infection of DCs was required to induce MVA-specific CD8+ T cell responses *via* both direct- and cross-presentation of viral antigens (33).

A main finding of our study was that APC activation involves the STING pathway. This was evidenced both through CXCL10 production by ORFV-infected DCs and the lack of activation of THP1-Dual KO STING cells. STING is a well-characterized adaptor molecule relevant for the cellular response to self and foreign cytosolic dsDNA (34). After detection of cytosolic dsDNA by cGAMP it binds to STING and induces IFN gene transcription (35). This is why STING-dependent signaling has been shown central for the induction of both innate and adaptive immunity in response to dsDNA viruses (36, 37) and was found to be critically involved in the induction of type I IFN induction in cDCs by MVA as well as a robust cytotoxic lymphocyte response connected with an MVA-based vaccination (36). However, we cannot exclude that activation of APCs by ORFV also occurs *via* additional pathways. Whereas MyD88 does not play a role in the activation of THP1-Dual cells in our experiment, Siegemund et al. showed that the activation of plasmacytoid bone-marrow derived dendritic cells (BM-pDCs) is dependent on this adaptor protein (31). Von Buttlar et al. further identified the endosomal receptor TLR9 as an ORFV-sensing receptor in BM-pDCs (32). To what extent ORFV is also recognized by TLR9 in human APCs remains to be determined by future experiments.

Initially, MVA infections were known to be recognized not only by DNA sensing pathways but also by TLR-dependent and -independent signaling pathways, including the RNA sensor melanoma differentiation-associated protein (MDA)-5, TLR2/TLR6 and NALP3 (38–42). In addition to cGAS, the STING-independent DNA sensors DNA-dependent protein kinase (DNA-PK) and Gamma-interferon-inducible protein (IFI16) are involved in immune activation by MVA (42) and therefore represent potential DNA sensors recognizing ORFV.

ORFV-based recombinant vectors have been successfully used in the past to develop prophylactic vaccines. A strong, long-lasting humoral immune response was elicited and protection against various infectious diseases was achieved in a wide range of animal species (11, 13–15, 43). However, whether and to what extent cellular immune responses are induced was not subject of these studies. Anyhow, depletion of CD8+ T cells after immunization with ORFV had no negative effect on protection against rabies or influenza virus infections (11, 13). In contrast, Rohde et al. suggested a T-cell-based protection of rabbits against infection with rabbit hemorrhagic disease virus, but without direct evidence for antigen-specific T cells (43). Schneider et al. recently demonstrated that an ORFV-based antitumor vaccine significantly inhibited tumor growth through inducing robust cellular immune responses, connected with complete tumor regression in rabbits after repeated administrations (24). Importantly, repeated vaccination with ORFV D1701-VrV did not induce ORFV-specific CD8+ T cell responses but induced respective immune responses towards an encoded transgene (12). In this study, the activation of both memory T cells and naïve T cells against ORFV-encoded transgenes was successfully demonstrated. This is in line with results from a very recently published proof-of-concept study of a D1701-VrV-based dengue virus (DENV) vaccine candidate (17).

However, our study has also some limitations. *In vitro* experiments with human PBMCs are subject to inherent variability, as these were obtained from a limited number of donors and such sample materials usually show considerable variance. Hence, the precise mechanisms of ORFV uptake and activation in tissue-resident APCs may differ and cannot be extrapolated from our data with absolute security. Although ORFV uptake by phagocytosis and macropinocytosis was established, a more detailed understanding of the molecular mechanisms would be desirable. In addition, although it has been shown that ORFV-mediated DC activation involves the STING pathway, the specific signaling cascade remains to be elucidated also in primary cells, as well as the potential role of other signaling pathways.

In conclusion, we successfully demonstrated that ORFV-mediated cellular immune responses involve virus uptake by APCs and their subsequent activation *via* the STING pathway. Activated APCs subsequently activate surrounding T cells, B cells, and NK cells. Furthermore, ORFV-infected DCs prime naïve T cells against encoded antigens and induce a potent recall response. These results support that ORFV is a promising viral vector for the induction of strong adaptive immune responses towards encoded antigens.

## Data Availability Statement

The original contributions presented in the study are included in the article/supplementary material. Further inquiries can be directed to the corresponding authors.

## Ethics Statement

The use of human biomaterials was reviewed and approved by the Ethics Committee of the Medical Faculty of Eberhard Karls University and the University Hospital of Tübingen (project number: 507/2017B01). All blood donors provided their written informed consent before study participation and use of their biomaterials.

## Author Contributions

Conceptualization, MM, AR, and RA. Methodology, MM and AR. Validation, MM, AR, and RA. Formal analysis, MM and AR. Investigation, MM and AR. Resources, RA. Data curation, MM, AR, and RA. Writing—original draft preparation, MM and AR. Writing—review and editing, MWL and RA. Visualization, MM, AR, and MWL. Supervision, MWL and RA. Project administration, RA and MWL. Funding acquisition, RA. All authors have read and agreed to the final version of the manuscript.

## Funding

This research was supported in part by the Institutional Strategy of the University of Tübingen (Deutsche Forschungsgemeinschaft ZUK63), the EXIST Forschungstransfer of the German Ministry for Economic Affairs and Energy, which is co-financed by the European Social Fund. We acknowledge support by Open Access Publishing Fund of the University of Tübingen.

## Conflict of Interest

RA is the inventor of patents on ORFV. RA and MM have ownership interest in Prime Vector Technologies GmbH. MWL is an inventor of patents owned by Immatics Biotechnologies and has acted as a paid consultant in cancer immunology for Boehringer Ingelheim Pharma & Co. KG.

The remaining authors declare that the research was conducted in the absence of any commercial or financial relationships that could be construed as a potential conflict of interest.

## Publisher’s Note

All claims expressed in this article are solely those of the authors and do not necessarily represent those of their affiliated organizations, or those of the publisher, the editors and the reviewers. Any product that may be evaluated in this article, or claim that may be made by its manufacturer, is not guaranteed or endorsed by the publisher.

## References

[1] Wang R Wang Y Liu F Luo S. Orf Virus: A Promising New Therapeutic Agent. Rev Med Virol (2019) 29(1):e2013. doi: 10.1002/jmv.25282 30370570

[2] Mercer AA Fraser K Barns G Robinson AJ. The Structure and Cloning of Orf Virus DNA. Virology (1987) 157(1):1–12. doi: 10.1016/0042-6822(87)90307-2 3029950

[3] Al-Salam S Nowotny N Sohail MR Kolodziejek J Berger TG. Ecthyma Contagiosum (Orf)–Report of a Human Case From the United Arab Emirates and Review of the Literature. J Cutan Pathol (2008) 35(6):603–7. doi: 10.1111/j.1600-0560.2007.00857.x 18201239

[4] Estela Cubells JR Braverman I Kashgarian M Lazova R. A 65-Year-Old Female From Connecticut With Orf Infection. Dermatopathology (2016) 3(2):55–60. doi: 10.1159/000447125 27504446 PMC4945808

[5] Mayr A Herlyn M Mahnel H Danco A Zach A Bostedt H. Control of Ecthyma Contagiosum (Pustular Dermatitis) of Sheep With a New Parenteral Cell Culture Live Vaccine. Zentralbl Veterinarmed B (1981) 28(7):535–52. doi: 10.1111/j.1439-0450.1981.tb01772.x 7331595

[6] Rziha HJ Büttner M Müller M Salomon F Reguzova A Laible D. Genomic Characterization of Orf Virus Strain D1701-V (Parapoxvirus) and Development of Novel Sites for Multiple Transgene Expression. Viruses (2019) 11(2):E127. doi: 10.3390/v11020127 30704093 PMC6409557

[7] Büttner M Rziha HJ. Parapoxviruses: From the Lesion to the Viral Genome. J Vet Med B Infect Dis Vet Public Health (2002) 49(1):7–16. doi: 10.1046/j.1439-0450.2002.00539.x 11911596

[8] Rziha H Henkel M Cottone R Bauer B Auge U Götz F. Generation of Recombinant Parapoxviruses: Non-Essential Genes Suitable for Insertion and Expression of Foreign Genes. J Biotechnol (2000) 83(1–2):137–45. doi: 10.1016/S0168-1656(00)00307-2 11000469

[9] Rziha HJ Henkel M Cottone R Meyer M Dehio C Büttner M. Parapoxviruses: Potential Alternative Vectors for Directing the Immune Response in Permissive and Non-Permissive Hosts. J Biotechnol (1999) 73(2–3):235–42. doi: 10.1016/S0168-1656(99)00141-8 10486932

[10] Rziha HJ Rohde J Amann R. Generation and Selection of Orf Virus (ORFV) Recombinants. Methods Mol Biol (2016) 1349:177–200. doi: 10.1007/978-1-4939-3008-1_12 26458837

[11] Amann R Rohde J Wulle U Conlee D Raue R Martinon O. A New Rabies Vaccine Based on a Recombinant ORF Virus (Parapoxvirus) Expressing the Rabies Virus Glycoprotein. J Virol (2013) 87(3):1618–30. doi: 10.1128/JVI.02470-12 PMC3554190 23175365

[12] Reguzova A Ghosh M Müller M Rziha HJ Amann R. Orf Virus-Based Vaccine Vector D1701-V Induces Strong CD8+ T Cell Response Against the Transgene But Not Against ORFV-Derived Epitopes. Vaccines (2020) 8(2):E295. doi: 10.3390/vaccines8020295 32531997 PMC7349966

[13] Rohde J Amann R Rziha HJ. New Orf Virus (Parapoxvirus) Recombinant Expressing H5 Hemagglutinin Protects Mice Against H5N1 and H1N1 Influenza A Virus. PloS One (2013) 8(12):e83802. doi: 10.1371/journal.pone.0083802 24376753 PMC3869816

[14] van Rooij EMA Rijsewijk FAM Moonen-Leusen HW Bianchi ATJ Rziha HJ. Comparison of Different Prime-Boost Regimes With DNA and Recombinant Orf Virus Based Vaccines Expressing Glycoprotein D of Pseudorabies Virus in Pigs. Vaccine (2010) 28(7):1808–13. doi: 10.1016/j.vaccine.2009.12.004 20018271

[15] Henkel M Planz O Fischer T Stitz L Rziha HJ. Prevention of Virus Persistence and Protection Against Immunopathology After Borna Disease Virus Infection of the Brain by a Novel Orf Virus Recombinant. J Virol (2005) 79(1):314–25. doi: 10.1128/JVI.79.1.314-325.2005 PMC538698 15596826

[16] Voigt H Merant C Wienhold D Braun A Hutet E Le Potier MF. Efficient Priming Against Classical Swine Fever With a Safe Glycoprotein E2 Expressing Orf Virus Recombinant (ORFV VrV-E2). Vaccine (2007) 25(31):5915–26. doi: 10.1016/j.vaccine.2007.05.035 17600594

[17] Reguzova A Fischer N Müller M Salomon F Jaenisch T Amann R. A Novel Orf Virus D1701-VrV-Based Dengue Virus (DENV) Vaccine Candidate Expressing HLA-Specific T Cell Epitopes: A Proof-Of-Concept Study. Biomedicines (2021) 9(12):1862. doi: 10.3390/biomedicines9121862 34944678 PMC8698572

[18] Mercer J Helenius A. Virus Entry by Macropinocytosis. Nat Cell Biol (2009) 11(5):510–20. doi: 10.1038/ncb0509-510 19404330

[19] Quemin ER Corroyer-Dulmont S Krijnse-Locker J. Entry and Disassembly of Large DNA Viruses: Electron Microscopy Leads the Way. J Mol Biol (2018) 430(12):1714–24. doi: 10.1016/j.jmb.2018.04.019 29702107

[20] Sallusto F Cella M Danieli C Lanzavecchia A. Dendritic Cells Use Macropinocytosis and the Mannose Receptor to Concentrate Macromolecules in the Major Histocompatibility Complex Class II Compartment: Downregulation by Cytokines and Bacterial Products. J Exp Med (1995) 182(2):389–400. doi: 10.1084/jem.182.2.389 7629501 PMC2192110

[21] Sarkar K Kruhlak MJ Erlandsen SL Shaw S. Selective Inhibition by Rottlerin of Macropinocytosis in Monocyte-Derived Dendritic Cells. Immunology (2005) 116(4):513–24. doi: 10.1111/j.1365-2567.2005.02253.x PMC1802442 16313365

[22] Norbury CC Chambers BJ Prescott AR Ljunggren HG Watts C. Constitutive Macropinocytosis Allows TAP-Dependent Major Histocompatibility Complex Class I Presentation of Exogenous Soluble Antigen by Bone Marrow-Derived Dendritic Cells. Eur J Immunol (1997) 27(1):280–8. doi: 10.1002/eji.1830270141 9022030

[23] Randolph GJ Beaulieu S Lebecque S Steinman RM Muller WA. Differentiation of Monocytes Into Dendritic Cells in a Model of Transendothelial Trafficking. Science (1998) 282(5388):480–3. doi: 10.1126/science.282.5388.480 9774276

[24] Schneider M Müller M Yigitliler A Xi J Simon C Feger T. Orf Virus-Based Therapeutic Vaccine for Treatment of Papillomavirus-Induced Tumors. J Virol (2020) 94(15):e00398-20. doi: 10.1128/JVI.00398-20 32404527 PMC7375371

[25] Flechsig C Suezer Y Kapp M Tan SM Löffler J Sutter G. Uptake of Antigens From Modified Vaccinia Ankara Virus-Infected Leukocytes Enhances the Immunostimulatory Capacity of Dendritic Cells. Cytotherapy (2011) 13(6):739–52. doi: 10.3109/14653249.2010.549123 21250864

[26] Altenburg AF van de Sandt CE Li BWS MacLoughlin RJ Fouchier RAM van Amerongen G. Modified Vaccinia Virus Ankara Preferentially Targets Antigen Presenting Cells *In Vitro*, Ex Vivo and *In Vivo* . Sci Rep (2017) 7(1):8580. doi: 10.1038/s41598-017-08719-y 28819261 PMC5561217

[27] Rziha HJ Büttner M. Parapoxviruses (Poxviridae). In: Bamford D. H. Zuckerman M, editors. Encyclopedia of Virology. Amsterdam, Oxford, Cambridge MA: Elsevier (2021). p. 666–74. Available at: https://linkinghub.elsevier.com/retrieve/pii/B9780128145159000588.

[28] Kieser Q Noyce RS Shenouda M James Lin YC Evans DH. Cytoplasmic Factories, Virus Assembly, and DNA Replication Kinetics Collectively Constrain the Formation of Poxvirus Recombinants. PloS One (2020) 15(1):e0228028. doi: 10.1371/journal.pone.0228028 31945138 PMC6964908

[29] Sandgren KJ Wilkinson J Miranda-Saksena M McInerney GM Byth-Wilson K Robinson PJ. A Differential Role for Macropinocytosis in Mediating Entry of the Two Forms of Vaccinia Virus Into Dendritic Cells. PloS Pathog (2010) 6(4):e1000866. doi: 10.1371/journal.ppat.1000866 20421949 PMC2858709

[30] Dutta D Donaldson JG. Search for Inhibitors of Endocytosis: Intended Specificity and Unintended Consequences. Cell Logist (2012) 2(4):203–8. doi: 10.4161/cl.23967 PMC3607622 23538558

[31] Siegemund S Hartl A von Buttlar H Dautel F Raue R Freudenberg MA. Conventional Bone Marrow-Derived Dendritic Cells Contribute to Toll-Like Receptor-Independent Production of Alpha/Beta Interferon in Response to Inactivated Parapoxvirus Ovis. J Virol (2009) 83(18):9411–22. doi: 10.1128/JVI.02362-08 PMC2738253 19570869

[32] von Buttlar H Siegemund S Büttner M Alber G. Identification of Toll-Like Receptor 9 as Parapoxvirus Ovis-Sensing Receptor in Plasmacytoid Dendritic Cells. PloS One (2014) 9(8):e106188. doi: 10.1371/journal.pone.0106188 25171368 PMC4149514

[33] Liu L Chavan R Feinberg MB. Dendritic Cells are Preferentially Targeted Among Hematolymphocytes by Modified Vaccinia Virus Ankara and Play a Key Role in the Induction of Virus-Specific T Cell Responses *In Vivo* . BMC Immunol (2008) 9:15. doi: 10.1186/1471-2172-9-15 18412969 PMC2359732

[34] Xiao TS Fitzgerald KA. The cGAS-STING Pathway for DNA Sensing. Mol Cell (2013) 51(2):135–9. doi: 10.1016/j.molcel.2013.07.004 PMC3782533 23870141

[35] Wu J Sun L Chen X Du F Shi H Chen C. Cyclic GMP-AMP Is an Endogenous Second Messenger in Innate Immune Signaling by Cytosolic DNA. Science (2013) 339(6121):826–30. doi: 10.1126/science.1229963 PMC3855410 23258412

[36] Georgana I Sumner RP Towers GJ Maluquer de Motes C. Virulent Poxviruses Inhibit DNA Sensing by Preventing STING Activation. J Virol (2018) 92(10):e02145-17. doi: 10.1128/JVI.02145-17 29491158 PMC5923072

[37] Lam E Stein S Falck-Pedersen E. Adenovirus Detection by the cGAS/STING/TBK1 DNA Sensing Cascade. J Virol (2014) 88(2):974–81. doi: 10.1128/JVI.02702-13 PMC3911663 24198409

[38] Guerra S Nájera JL González JM López-Fernández LA Climent N Gatell JM. Distinct Gene Expression Profiling After Infection of Immature Human Monocyte-Derived Dendritic Cells by the Attenuated Poxvirus Vectors MVA and NYVAC. J Virol (2007) 81(16):8707–21. doi: 10.1128/JVI.00444-07 PMC1951336 17537851

[39] Barbalat R Lau L Locksley RM Barton GM. Toll-Like Receptor 2 on Inflammatory Monocytes Induces Type I Interferon in Response to Viral But Not Bacterial Ligands. Nat Immunol (2009) 10(11):1200–7. doi: 10.1038/ni.1792 PMC2821672 19801985

[40] Zhu J Martinez J Huang X Yang Y. Innate Immunity Against Vaccinia Virus Is Mediated by TLR2 and Requires TLR-Independent Production of IFN-Beta. Blood (2007) 109(2):619–25. doi: 10.1182/blood-2006-06-027136 PMC1785085 16973959

[41] Andrejeva J Childs KS Young DF Carlos TS Stock N Goodbourn S. The V Proteins of Paramyxoviruses Bind the IFN-Inducible RNA Helicase, Mda-5, and Inhibit its Activation of the IFN-Beta Promoter. Proc Natl Acad Sci USA (2004) 101(49):17264–9. doi: 10.1073/pnas.0407639101 PMC535396 15563593

[42] El-Jesr M Teir M Maluquer de Motes C. Vaccinia Virus Activation and Antagonism of Cytosolic DNA Sensing. Front Immunol (2020) 11:568412. doi: 10.3389/fimmu.2020.568412 33117352 PMC7559579

[43] Rohde J Schirrmeier H Granzow H Rziha HJ. A New Recombinant Orf Virus (ORFV, Parapoxvirus) Protects Rabbits Against Lethal Infection With Rabbit Hemorrhagic Disease Virus (RHDV). Vaccine (2011) 29(49):9256–64. doi: 10.1016/j.vaccine.2011.09.121 22001119

